# Equally able, but unequally accepted: Gender differentials and experiences of community health volunteers promoting maternal, newborn, and child health in Morogoro Region, Tanzania

**DOI:** 10.1186/s12939-015-0201-z

**Published:** 2015-08-25

**Authors:** Isabelle Feldhaus, Marissa Silverman, Amnesty E. LeFevre, Rose Mpembeni, Idda Mosha, Dereck Chitama, Diwakar Mohan, Joy J. Chebet, David Urassa, Charles Kilewo, Marya Plotkin, Giulia Besana, Helen Semu, Abdullah H. Baqui, Peter J. Winch, Japhet Killewo, Asha S. George

**Affiliations:** Johns Hopkins Bloomberg School of Public Health, 615 North Wolfe Street, Baltimore, MD 21205 USA; Muhimbili University of Health and Allied Sciences, United Nations Road, 65001 Dar es Salaam, Tanzania; Jhpiego, 1615 Thames Street, Baltimore, MD 21231 USA; Ministry of Health and Social Welfare, 6 Samora Machel Avenue, 11478 Dar es Salaam, Tanzania; Johns Hopkins Bloomberg School of Public Health, c/o MUHAS, United Nations Road, 65001 Dar es Salaam, Tanzania

## Abstract

**Background:**

Despite emerging qualitative evidence of gendered community health worker (CHW) experience, few quantitative studies examine CHW gender differentials. The launch of a maternal, newborn, and child health (MNCH) CHW cadre in Morogoro Region, Tanzania enlisting both males and females as CHWs, provides an opportunity to examine potential gender differences in CHW knowledge, health promotion activities and client acceptability.

**Methods:**

All CHWs who received training from the Integrated MNCH Program between December 2012 and July 2013 in five districts were surveyed and information on health promotion activities undertaken drawn from their registers. CHW socio-demographic characteristics, knowledge, and health promotion activities were analyzed through bi- and multivariate analyses. Composite scores generated across ten knowledge domains were used in ordered logistic regression models to estimate relationships between knowledge scores and predictor variables. Thematic analysis was also undertaken on 60 purposively sampled semi-structured interviews with CHWs, their supervisors, community leaders, and health committee members in 12 villages from three districts.

**Results:**

Of all CHWs trained, 97 % were interviewed (*n* = 228): 55 % male and 45 % female. No significant differences were observed in knowledge by gender after controlling for age, education, date of training, marital status, and assets. Differences in number of home visits and community health education meetings were also not significant by gender. With regards to acceptability, women were more likely to disclose pregnancies earlier to female CHWs, than male CHWs. Men were more comfortable discussing sexual and reproductive concerns with male, than female CHWs. In some cases, CHW home visits were viewed as potentially being for ulterior or adulterous motives, so trust by families had to be built. Respondents reported that working as female–male pairs helped to address some of these dynamics.

**Conclusions:**

Male and female CHWs in this study have largely similar knowledge and health promotion outputs, but challenges in acceptance of CHW counseling for reproductive health and home visits by unaccompanied CHWs varied by gender. Programs that pair male and female CHWs may potentially overcome gender issues in CHW acceptance, especially if they change gender norms rather than solely accommodate gender preferences.

## Background

There is renewed interest in community health worker (CHW) programs globally, particularly in light of evidence that CHWs can effectively implement interventions that reduce mortality and morbidity among women and children when compared to facility-based services alone [1–6]. In 2012, the World Health Organization (WHO) published recommendations on task shifting to improve maternal, newborn, and child health (MNCH), strongly suggesting that lay health workers be used for health promotion, education, and continuous support for women before, during, and after labor [7]. With strengthened health systems, appropriate training, supervision, and remuneration, CHWs may serve an integral role in improving child survival [8], and maternal and child health more broadly.

CHWs are relevant to Tanzania when considering the health provider shortages and maldistributions that stymie service delivery at all levels impeding the effective implementation of MNCH interventions [9, 10]. When compared to the WHO-recommended health workforce density of 25 health professionals (including physicians, nurses, and midwives) per 10,000 people, Tanzania lags behind with only four health professionals for every 10,000 citizens [11, 12]. Although nearly three quarters of Tanzanians live in rural areas, only a third of doctors practice in rural areas [13]. Mirroring these inequalities, 81 % of urban women have a skilled birth attendant during delivery, while only 38 % of rural women do [14].

In recognition of these health system challenges and as complications in childbirth and pregnancy remain the leading causes of death among women of reproductive age, the Tanzanian Ministry of Health and Social Welfare (MOHSW) increased focus on MNCH care [15], especially at community levels [16]. Following these policy recommendations, an MNCH-focused CHW program was piloted by the MOHSW in Morogoro Region with support from the USAID-funded Mothers and Infant, Safe, Healthy and Alive Program (MAISHA) program. In this pilot program, CHWs were trained and deployed to conduct a census of the population in their catchment area; identify and monitor pregnant and postpartum women, as well as newborns and children under 5 years of age; and provide health education and behavior change messages to all pregnant and postpartum women, their partners, and the community as a whole. Counseling and health education messages encouraged pregnancy care, birth preparedness, safe delivery practices and newborn care, and maternal and child nutrition, among other MNCH topics. The guidelines supporting the CHW program also recognized gender and gender-based violence as a barrier to service utilization and tasked CHWs with encouraging male engagement and involvement in birth preparedness, family planning, and couple testing for HIV.

As the Integrated MNCH Program was designed to deploy both male and female CHWs, an opportunity arose to examine potential differences in the knowledge and experience of CHWs by gender. Currently, limited evidence exists on the role of gender on CHW knowledge, health activities, and MNCH outcomes [6]. Quantitative and qualitative evaluations of CHW programs rarely differentiate their analysis by sex to reveal differences or similarities in CHW experiences [17]. Yet gender not only has a clear impact on reproductive, maternal, and child health [18], but also across human resources for health [17, 19, 20].

It is at times assumed that female CHWs are better positioned than male CHWs to achieve improved health outcomes for women and children. For example, the lack of acceptance of male lay health workers by pregnant women was reported to contribute to their low impact on maternal health in Nigeria [21]. Similarly, in Somalia, male CHWs had considerable challenges effectively reaching and providing health counseling to women, especially for reproductive health issues [22]. In rural Afghanistan, the presence of a female CHW in the community was associated with higher utilization of reproductive health services, including antenatal care and skilled birth attendants, while the presence of a male CHW was not [23].

At the same time, relying exclusively on female staff can also consolidate existing conservative gender norms and narrowly focus on women as the target of maternal and child health programs, excluding engagement with men and other senior family members [17, 24]. It may also ignore strengths and variability that may differ by sex among CHWs. In Kenya, findings from a CHW program conducting home visits during pregnancy, showed that male CHWs were 1.6 times more likely than female CHWs to keep complete client records, and 71 % more likely to provide consultations resulting in favorable client behavior change, while female CHWs were 58 % more likely to counsel their clients correctly compared to their male counterparts [25].

As Tanzania moves towards formalizing a CHW cadre and scaling up the CHW program, information is needed regarding the strengths, weaknesses, and contextual factors surrounding performance of this new cadre [26]. Among these are performance-related factors associated with CHW gender. This study investigates differences and similarities among male and female MNCH CHWs in rural Tanzania in regards to knowledge, health promotion activities, and acceptance by the community.

## Methods

### Setting

With a population of over 49.6 million people, Tanzania is the fifth most populous country in Sub-Saharan Africa [27], and has nearly 70 % of its people living below the international poverty line of $1.25 PPP a day [28]. In 2010, the country reported an infant mortality rate of 51 deaths per 1000 live births and an under-five mortality rate of 81 deaths per 1000 live births [14, 29, 30]. While child mortality has declined since 1990, Tanzania still has one of the highest maternal mortality rates in the world at 454 deaths per 100,000 live births [14, 27, 30].

Morogoro Region, often referred to as the “bread basket” of Tanzania, sits 200 km southwest of Dar es Salaam and is home to over 2.2 million people across 70,000 km^2^ [14]. Although regional averages for education and poverty are similar to national averages, overall health outcomes are somewhat higher [14]. Women aged 15–49 years in Morogoro report more use of skilled birth attendants (61 % versus 51 %), more facility deliveries (58 % versus 50 %), and fewer barriers to accessing care (23 % versus 36 %) compared to national averages [14]. Children under five in Morogoro are more likely to have been taken to a facility when presenting with a fever (96 % versus 65 %), and children 12–23 months are more likely to have a vaccination card (96 % versus 84 %) compared to national averages [14].

### Integrated MNCH Program recruitment & training

Male and female volunteers, selected by their village leaders, with ideally a minimum of secondary (form 4) education and who resided in the village of service, were trained on maternal, newborn and child health topics for 21 days. Training methodology included classroom-based sessions and a few days of accompanied field visits seeing clients with their trainers. Following training and an evaluative post-test, trainees were awarded the title of MNCH CHWs and provided with a learning reference manual, job aids, and client registers. Guidelines and job aids outlined topics to be covered over the course of different periods of time, ranging from seven to ten MNCH topics for each home visit. Upon returning to their home villages after training, village leaders introduced CHWs to the community to facilitate their work. All CHW were also assigned to a health facility supervisor who would support them at the community level.

### Sampling

A total of 238 MNCH CHWs were eligible for inclusion in the study, having been trained under the Integrated MNCH Program at least three months prior to data collection. For the quantitative component of the study, a face-to-face structured questionnaire was administered in October 2013 to 228 of the 238 MNCH CHWs trained. Participants were from five of Morogoro’s six districts: Morogoro Rural, Mvomero, Kilosa, Gairo, and Ulanga. Participants were not included if they did not consent to the interview (*n* = 0), dropped out of the program (*n* = 3), were travelling with an unknown return date (*n* = 5), were sick or in the hospital (*n* = 1), or were deceased (*n* = 1) at the time of data collection. Assuming that composite knowledge scores within male and female CHWs was normally distributed with a standard deviation of 15 points, we would have been able to detect a difference of six points in mean scores between male and female CHWs with 80 % power and a type I error probability of 0.05.

A sub-sample of the total eligible MNCH CHW population was recruited for participation in the qualitative study in three of the five districts previously selected: Morogoro Rural, Kilosa, and Gairo Districts. Four health center catchment areas were selected in these districts, with CHWs drawn from one health center village, one dispensary village, and one village with no government health care facility within each of these catchment areas. Lists of CHW Supervisors, CHWs, Village Executive Officers (VEO), Village Chairpersons, and Health Committee Members in each of the twelve villages were drawn up, with three to seven potential respondents in each village interviewed. Study respondents were purposefully selected to fulfill diverse CHW stakeholder criteria and gender balance among those who were available on the day of the facility/village visit.

### Data collection

A team of experienced research assistants received one-week of training prior to data collection. Study personnel visited the provider “in-charge” of health facilities to obtain CHW contact information from facility-based supervisors. CHWs were then contacted either via mobile phone or through village leaders, but not through their supervisors. Consenting CHWs were interviewed in their homes to maintain privacy and directly observe socioeconomic status.

Interviews were conducted in Swahili and approximately took 1 h and 30 min. Questions covered socio-demographic characteristics, knowledge, training, supervision, remuneration, challenges, referrals to health facilities, satisfaction, motivation, recording, and reporting. Reported knowledge was assessed through 44 questions about pregnancy care, postpartum care for mothers and newborns, child health, nutrition, HIV, malaria, family planning, and infection and injury prevention. Though unprompted, respondents were permitted to use the job aids provided by the Integrated MNCH Program in answering the questionnaire. CHW registers were used to collect selected indicators reporting health promotion activities for five months prior to data collection, May to September 2013.

Completed surveys were transported to Dar es Salaam for data entry and cleaning every two weeks during data collection. Quantitative data were double-entered and cleaned using EpiInfo software at MUHAS in Dar es Salaam. Surveys that recorded free-text answers were translated from Swahili into English. Data entry and cleaning was completed by December 2013.

For the qualitative study, Tanzanian research assistants underwent one week of training in qualitative research methods, which included piloting and finalizing data collection tools. In October 2013, research assistants conducted semi-structured interviews with respondents in a private and convenient location of the respondent’s choosing. The interview guide covered a range of questions spanning their personal background and motivation to participate in the program, their views on supervision and other CHW support mechanisms, and a sub-section of questions probing on experiences related to the social profile of CHWs. A study supervisor conducted daily debriefing sessions with research assistants and a group mid-point debriefing session to triangulate findings, discuss emerging themes, and refine probing and research focus during interviews. All interviews were done in Swahili and voice recordings were uploaded to a study supervisor’s computer within one day of an interview and checked for completion and recording clarity. Each research assistant transcribed his or her own interviews in Swahili, which were later translated by Tanzanian translators based in Dar es Salaam into English and uploaded into ATLAS.ti (version 7).

### Data analysis

Statistical analyses were performed using Stata 12.0 in Baltimore, MD, USA. Exploratory data analyses revealed frequencies and trends across socio-demographic variables and health promotion activities. Using principal components analysis, an asset index denoting relative wealth was constructed from CHW household assets and categorized into quintiles [31]. CHWs were scored 1 and 0 based on their answers to the questions on knowledge across the various domains previously described. The mean score for each domain was derived by aggregating scores from all items in a particular domain and calculating an average score for the respective domains. An overall composite score was calculated by aggregating and averaging scores from all items across all domains. The knowledge scores attained by CHWs were treated as ordered categorical variables under the assumption that the levels of scores have a natural order (low to high; higher scores indicative of greater knowledge). Ordered logistic regression models were used to analyze association between knowledge scores and gender, controlling for age, education, marital status, date of training, and wealth quintiles. The ordered logit model depends upon the idea of the cumulative logit, or that of cumulative probability. The cumulative probability C_ij_ is the probability that the *i*^th^ CHW is in the *j*^th^ or higher category:

$$ {C}_{ij}= Pr\left({y}_i\le j\right)={\displaystyle \sum_{k= 1}^j Pr\left({y}_i=k\right)} $$

The odds ratio for gender is the odds that a female CHW will have a score higher than a male CHW, given that all other factors are equal. Additionally, mean frequencies per month by gender were calculated and plotted for key indicators collected by CHWs through their registers to examine any differences associated with health promotion outputs.

For the qualitative study, transcripts were coded using ATLAS.ti by investigators based in Baltimore. Investigators developed a codebook based on prior field debriefings and subsequent review and discussion of transcripts with investigators based in Dar es Salaam through weekly Skype calls. Thematic analysis included triangulating responses across different respondent types, examining common patterns, and exploring outliers or potential negative findings to understand nuances in the data. Analysis was further strengthened by sharing preliminary findings with other team members and other project partners, including the MOHSW Tanzania through preliminary and national workshops in Dar es Salaam.

This study received ethical approval from Muhimbili University of Health and Allied Sciences (MUHAS) and Johns Hopkins Bloomberg School of Public Health (JHSPH) Institutional Review Boards (IRB).

## Results

### CHW profile & gender

Of all CHWs trained for the Integrated MNCH Program, 97 % were interviewed: 55 % male and 45 % female. Male and female CHWs were similar with respect to dates of training, education, ability to read, languages spoken, number of dependents, and income characteristics. In terms of education, 54 and 50 % of male and female CHWs, respectively, had completed secondary (form 4) education (*p* = 0.558). Although male CHWs seemed to be older and more likely to be married than female CHWs, the only significantly different social characteristic distinguishing male and female CHWs, was that male CHWs (97 %) were more likely to report agricultural activities as their primary income-generating activity compared to females (90 %, *p* = 0.042) (Table 1).

**Table 1:** CHW profile and characteristics by gender

Characteristic	Male		Female		Chi-square
	(*n* = 125)	%	(*n* = 103)	%	
Date of training					
Dec 2012–Jan 2013	24	19 %	22	21 %	0.16
April–May 2013	49	39 %	37	36 %	0.26
July 2013	51	41 %	43	42 %	0.02
Age, years (mean/median/range)	33.6/32/20–61		32.4/32/19–59		-
<25 years	35	28 %	33	32 %	0.44
25–35 years	39	31 %	36	35 %	0.36
>35 years	51	41 %	34	33 %	1.47
Marital status					
Married	80	64 %	53	51 %	3.66
Not married	45	36 %	50	49 %	3.66
Education					
Partial primary	3	2 %	1	1 %	0.67
Primary or higher completed	121	97 %	99	96 %	0.08
Language					
Swahili	125	100 %	103	100 %	-
Regional dialect	103	82 %	89	86 %	0.68
English	21	17 %	13	13 %	0.73
Number of dependents (mean/median/range)	3.28/3/0–11		3.28/4/0–12		-
Income-generation					
Agriculture	121	97 %	93	90 %	4.15*
Non-agriculture	15	12 %	14	14 %	0.13
Household income per month, all sources (mean/median/range)	$45/$29/$0–274		$50/$30/$0–305		0.63

*p < 0.05

### CHW knowledge

**Fig. 1:**
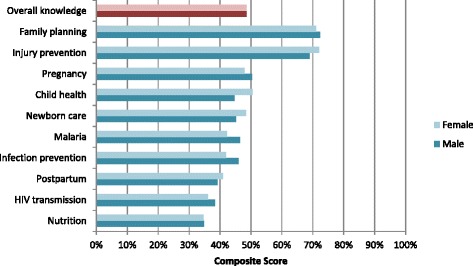
Mean composite scores for CHW knowledge and type of service provision by gender

Male and female CHWs received similar composite scores of 48.6 and 48.5 % respectively for overall MNCH knowledge (Fig. 1). Both men and women reported the highest knowledge surrounding the topic of family planning and the least around nutrition. Ordered logistic regression models of composite knowledge scores for female CHWs, also indicated no statistically significant differences by gender when adjusted for age, education, marital status, date of training, and wealth quintiles (Table 2). There were no significant differences in knowledge reported on specific subtopics (e.g., child health, postpartum and newborn care, nutrition, etc.) between female and male CHWs.

**Table 2:** Odds ratios of knowledge scores for female over male CHWs from ordered logistic regression models

Knowledge domain	Unadjusted OR (95 % CI)	Adjusted OR (95 % CI)
Overall knowledge	1.02 (0.64, 1.63)	1.07 (0.65, 1.75)
Child health	1.29 (0.50, 1.24)	1.19 (0.73, 1.96)
Postpartum	1.24 (0.78, 1.95)	1.22 (0.75, 1.99)
Newborn care	1.24 (0.78, 1.95)	1.16 (0.70, 1.90)
Injury prevention	1.24 (0.77, 1.98)	1.19 (0.71, 1.98)
Nutrition	1.05 (0.67, 1.66)	1.05 (0.65, 1.74)
Family planning	0.97 (0.61, 1.52)	0.99 (0.61, 1.60)
HIV	0.85 (0.54, 1.34)	0.88 (0.54, 1.44)
Pregnancy	0.79 (0.50, 1.24)	0.77 (0.47, 1.27)
Infection prevention	0.79 (0.50, 1.25)	0.75 (0.47, 1.20)
Malaria	0.68 (0.43, 1.09)	0.69 (0.42, 1.15)

Adjusted ORs controlled for date of training, education, age, marital status, and wealth quintile

### CHW activities

Male and female CHWs reported no significant differences in the types of CHW activities in which they engaged or the amount of hours and days spent doing them (Table 3). Both male and female CHWs reported working for a median of 3 days per week, a median of 4 h per day. While the median number of households CHWs reported serving within their catchment area varied for male (146) and female CHWs (100) this was not significantly different.

**Table 3:** Summary of reported CHW activities by gender

Activity	Male	Female	p-value
	(*n* = 125)	(*n* = 103)	
Days per week providing services (mean/median/range)	2.8/3/0–5	2.9/3/0–7	0.63
Hours worked per day providing services (mean/median/range)	5.0/4/1–12	4.7/4/1–11	0.40
Households served by Integrated Program CHW (mean/median/range)	197/146/3–1702	157/100/20–1500	0.18
Work in health facilities (n, %)	93 (74.4)	69 (67.0)	0.22
Work with other CHW programs (n, %)	18 (14.4)	22 (21.4)	0.18
Distance from home to facility (km, mean/median/range)	5.2/3/1–50	4.1/3/0–20	0.16
Mode of transportation to health facility (n, %)			
Foot	87 (69.6)	77 (74.8)	0.39
Bicycle	34 (27.2)	17 (16.5)	0.05
Motorbike	6 (4.8)	12 (11.7)	0.06

*p < 0.05

Slightly more female CHWs (21.4 %) reported working for other CHW programs in addition to their commitment to the Integrated MNCH Program compared to male CHWs (14.4 %). More male CHWs (74.4 %) reported working with a health facility to support the provision of certain health services, (e.g., home follow-up of patients, weighing children, support providers during vaccination days, vitamin A campaigns, deworming campaigns, and recordkeeping) compared to female CHWs (67.0 %). Walking by foot was reported as the most common mode of transportation to the health facility for both male (69.6 %) and female CHWs (74.8 %), although men reported using a bicycle (27.2 %) more often than women (16.5 %), while women reported hiring a motorbike (11.7 %) more often than men (4.8 %). However, none of these gender differences were statistically significant.

**Fig. 2:**
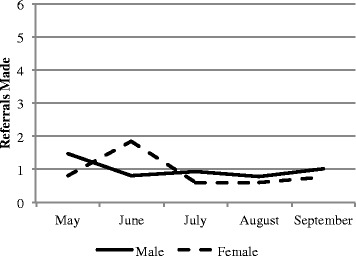
Mean referrals and household visits made by CHW by gender, May-September 2013

**Fig. 3:**
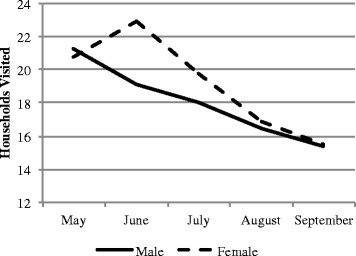
Mean household visits made by CHWs by gender, May-September 2013

Health promotion output data extracted from the registers maintained by CHWs revealed similar trends among male and female CHWs (Fig. 2 & 3 ). Among both male and female CHWs, the mean number of visits per CHW decreased over time, as the number of CHWs in the catchment area increased. The total number of home visits, referrals, health education meetings and people attending those meetings did not differ significantly for either gender.

### Respondent profile for qualitative study

In total, 60 respondents were interviewed (Table 4), including 8 CHW facility-based supervisors, 15 CHWs, 15 village leaders, and 18 health committee members from 12 villages. While gender balance was achieved across most respondent types, village leaders were primarily male.

**Table 4:** Participants in qualitative study on CHW experiences and support systems

Respondent type	Male, n	Female, n	Total
CHW	9	6	15
Village leader	14	1	15
Health committee member from the community	11	7	18
Health provider who supervises CHW	5	7	12
Total	39	21	60

When research assistants initially asked respondents if they felt that being a woman or a man affected their job in the community, nearly all respondents, regardless of their gender, initially responded ‘no.’ With probing, village leaders almost unanimously insisted that gender does not affect a CHW’s work, while nearly all other respondents, including CHW themselves, reported that challenges and advantages exist for male and female CHWs due to their gender.

### Gender and communication with CHWs

First, respondents noted that their MNCH work entails sex-specific issues that are best initially addressed by either female or male CHWs. Female CHWs are best suited to respond to the needs of pregnancy and other reproductive health issues among women in the village, as male CHWs generally find it difficult to identify or discuss pregnancy with women.

*“I ask a woman if there is any problem, she cannot hide it because we are of the same gender so we cooperate together”* (CHW, female, 3701)

*“…because women hide some things, you might ask her a question then she hides, you might ask her how many months is your pregnancy, she may tell you 3 months while it is 4 months, if it were her fellow woman she would tell her openly”* (CHW, male, 1201_B)

Likewise, female CHWs believed that it would be difficult to talk with men about reproductive health matters, and that men may prefer to receive reproductive health information from male CHWs:

“*There are things which they may hesitate to divulge to me such as mentioning problems with their private parts”* (CHW, female, 1801)

*“Some may say they would like their fellow men to come and talk to them and not a woman”* (CHW, female, 2401)

### Gender and access to households by CHWs

CHWs were not automatically accepted by the community, despite being from the community and selected by its members. In particular, they had to build trust with regard to home visits. CHW respondents explicitly mentioned or alluded to difficulties male and female CHWs face in making home visits, as their motivations were initially seen to be ulterior or misconstrued as adulterous. Overtime, as communities become more familiar with CHWs and their work, these misconceptions were reported to change.

“*…when you visit mothers of some families, they may doubt that you have gone there for other issues and not the health issues. Others are jealous because they think that we are after their husbands”* (CHW, female, 3301)

*“When we started, they were saying, ‘If someone comes to visit my wife, I will say he has committed adultery’. First people were having wrong idea…but as we continue to work… they start to trust us. Now we are recognized. Now if you go to someone’s house there is no problem because they know him”* (CHW, male, 3701)

“*If a female goes to a woman it’s not a problem, but you males need to be careful”* (Village Leader, male, 5303)

CHW supervisors understood the gendered issues surrounding community acceptance of CHWs and in particular, home visits with pregnant women by male CHWs.

*“…men do not like their wives to be visited when they are not around, so we teach them that they should be two…”* (CHW Supervisor, male, 8607)

### Working in pairs

One strategy to address the gender issues regarding CHW acceptability that emerged from respondents was to have both a male and female CHW in one community, or encouraging male–female CHW pairs to work together.

*“Gender balance simplifies the implementation of duties of village health workers. There are some sensitive questions which necessarily need a woman to communicate with her fellow women. Also, there are some sensitive questions which necessarily need a man to communicate with his fellow men. For instance, questions about toilet cleanness when asked by a man to a woman, inconveniencies are likely to occur”* (Health Committee Member, male, 5305)

The paired strategy was also presented as a solution to CHWs tasked with following up with their own relatives or members of the community with whom they had particularly close relationships. Cultural norms dictate that male relatives should not discuss reproductive health matters with their female relatives or kin, and vice versa. Having a CHW colleague of the opposite sex may facilitate CHW counselling on sex specific sensitive topics particularly linked to sexual and reproductive health.

*“One of the challenges is that because the education is about private matters, we do exchange positions. For example, I cannot tell my own mother about bad signs of pregnancy like bleeding, etc.…instead, I ask my colleague to come and talk to them. Talking to my mother on issues of genital discharge can be seen as immoral in our society”* (CHW, male, 8601)

Respondents acknowledged that women are generally responsible for reproductive or family health, but that men should also be involved. Male CHWs were seen to be critical in reaching out to husbands, particularly in the case of polygamous households. More generally, male–female pairs were seen as useful in educating husbands and wives, both separately and together.

*“…this is not only a problem for pastoralists, but even the other tribes have a tendency of staying aside and leaving all the matters related to mother and child health to the women. … Therefore when I provide services to the pregnant women, I always tell the men that they are also supposed to participate”* (CHW, male, 8501)

*“So I called them and told them, when you go to a certain family don’t call the mother alone, call the father too, so the husband can also hear the questions”* (Village Leader, male, 5303)

## Discussion

Within the context of a new MNCH CHW program in Morogoro Region, Tanzania, we found that respondents did not initially report gender to be an issue for CHWs. Male and female CHWs are largely similar in terms of demographic profile, knowledge retention from training, and provision of health promotion activities. While not statistically significant, slight differences in CHW knowledge and activities by gender may speak to underlying contextual influences on CHW performance and effectiveness in rural Tanzanian communities.

While no significant differences were found in CHW knowledge or amount of health promotion activities undertaken, qualitative data illustrated that gender did influence CHW acceptability. Respondents reported that CHW gender affected women’s disclosure and discussions about their pregnancy, and both male and female discomfort with discussing sexual and reproductive topics with the opposite sex. Both male and female CHWs also reported experiencing mistrust from community members and accusations of adultery, although this perception was noted to change as the community becomes more familiar with CHWs and their duties. Our findings also suggest that pairing of male and female CHWs has potential to improve community acceptance of CHWs and support more effective communication with male and female family members. In doing so, CHWs tread a fine balance between respecting social preferences and engaging in interactions and conversations that lead to changing gender norms about what topics are acceptable to discuss with whom.

### Limitations

This evaluation of the Integrated MNCH Program took place relatively soon after CHW training and CHWs were allowed to refer to their job aids and reference manual during the interviews, although few did so. The results therefore reflect short-term recall of health promotion content after training. Furthermore, what CHWs report through a structured interview with unprompted responses about their knowledge is different from what health promotion counseling messages they impart in practice with or without the use of their job aids and reference manual. Longitudinal assessments that assess knowledge over time are a critical gap in CHW performance studies [32]. Much of this study also relies on data provided by CHW registers, the quality and reliability of which have not yet been fully assessed since the initiation of the Integrated MNCH Program. The study took place within a few months of training and therefore reflects suggestive differences that were not significant but that could evolve over time.

Our qualitative exploratory analysis is colored by the type of respondents interviewed. While we triangulated findings across CHWs, supervisors, health committee members and village leaders, our analysis could have been further strengthened if beneficiaries of the CHW program had also been included. We also did not interview CHWs who were no longer working. These ‘drop-outs’ might have had experiences related to gender that prompted their attrition, although this was not raised as a reason for attrition by respondents interviewed. Interviews were also done during a short time period. However, the extended experience of co-investigators with the program and with MNCH in Tanzania helped to interpret and contextualize findings.

### Implications for MNCH CHW research and policy

This study builds on the currently limited scientific literature surrounding CHWs from a gender perspective, particularly in rural settings and for MNCH services [6, 17] and can in particular inform similar CHW programs in East Africa. As CHW ability did not seem to be differentiated by gender, but their acceptability was, male–female pairing may be an effective strategy to address these gender issues and provide greater support for male involvement in interventions for MNCH [33]. At the same time, male–female pairing of CHWs may not suit the needs of all target populations. It may be less appropriate for single adolescents or women who are pregnant from men who are not their husbands. It is also a more time intensive strategy, as CHWs could otherwise individually cover a higher number of households.

Nonetheless, male–female pairing of CHWs is one potential way to challenge current gender norms and support male engagement in MNCH in a transformative manner. With certain exceptions, such as in the case of women who have experienced violence from their male partners, there is general acceptance and desire for male involvement in MNCH, from both men and women. Emerging evidence also points to increased care-seeking behavior and improved health outcomes in certain contexts due to male engagement in MNCH. These efforts must be undertaken sensitively so as to respect women’s autonomy and to transform gender relations, rather than accommodate conservative gender norms that skew decision making to men exclusively [34]. Interventions such as the Women Centered Health Project in India provided male health workers with communication and counseling skills to talk about gender, sexuality, and sexual health issues with men and women in the community, but also enabled male workers to challenge their own conceptions of gender, sexuality, and other social constructs [33].

Tanzania is not alone in supporting male–female CHW cadres. In Rwanda, biomes (male–female CHW pairs) make household visits to men and women in the community [35]. Recent CHW program and policy recommendations support the use of male–female pairs of CHWs to travel and work together in communities, as well as share responsibilities between male and female CHWs [35]. It is important that the use of male–female CHW pairs be used to support men’s positive roles in women’s and children’s health, rather than further silo male health workers or the men who receive their services. For example, in Iran, male “*behvarzes,”* or CHWs, are primarily responsible for environment and sanitation projects, while female CHWs are tasked with MNCH duties [36]. Such stratification of female and male CHW tasks may reinforce conservative gender norms, rather than create a basis for progressive gender norms and advancing gender equity.

Evaluations of lay health worker programs have found that relying solely on women to fill these positions can be detrimental. For example, an analysis of a family planning lay health worker program in Indonesia found that by using only female volunteers, the program “had the effect of institutionalizing reproductive and family and community care roles and responsibilities within the sphere of women,” to the exclusion of men [37]. In Brazil, Portella and Gouveia found that reliance on community female health agents for maternal health services failed to address gender norms around men’s role in childcare and domestic violence, and men’s sexual behaviors that increased STI risk among their wives [38]. The assumption that female only CHW programs empower women must be challenged, especially if these programs are also unsupported and underfunded [17, 19]. Solely relying on female CHWs may replicate gender norms and biases in communities and within health systems, and leave power relations that require addressing male gendered behavior unaddressed [17, 19].

## Conclusion

Most national policies for lay health workers do not consider the role of gender in the development and implementation of these programs [39]. Yet gender remains an essential factor in relationships and dynamics influencing community level MNCH interventions, including CHW programs. In light of the scale-up of CHWs for MNCH programs in multiple settings, we must consider *who* will serve as the foundation for these health systems and *how* they will do so. Our research suggest that both male and female CHWs are equally able candidates for successful health promotion efforts, but that further attention is required to the role gender plays in community acceptability of MNCH. In the Tanzanian context, performing home visits in pairs seemed to mitigate gender barriers in providing MNCH services and encourage the engagement of men in MNCH. As community-based MNCH programs with lay health workers are scaled up in Tanzania and other countries, gender considerations should be addressed. These considerations have the potential to not only improve MNCH service delivery outcomes, but also may contribute to increasing gender equity among CHWs and in the community more broadly.

## Abbreviations

CHW: Community health worker
IRB: Institutional review board
JHSPH: Johns Hopkins school of public health
MNCH: Maternal, newborn, and child health
MOHSW: Ministry of health and social welfare
MUHAS: Muhimbili university of health and allied sciences
NGO: Non-governmental organization
VEO: Village executive officer
WHO: World Health Organization
